# Surgical Treatment of Brain Tumor-Related Epilepsy: Current and Emerging Strategies

**DOI:** 10.3390/cancers17213539

**Published:** 2025-11-01

**Authors:** Bobak F. Khalili, Michael R. Chojnacki, Karan Dixit, Kapil Gururangan, Craig Horbinski, Joshua M. Rosenow, Jason K. Hsieh, Stephen T. Magill, Matthew C. Tate, Rimas V. Lukas, Jessica W. Templer

**Affiliations:** 1 Rush Medical College, Rush University, Chicago, IL 60612, USA; bobak_khalili@rush.edu (B.F.K.); michael_chojnacki@rush.edu (M.R.C.); 2 Department of Neurology, Northwestern University, Chicago, IL 60611, USA; karan.dixit@nm.org (K.D.); kapil.gururangan@nm.org (K.G.); rimas.lukas@nm.org (R.V.L.); 3 Lou & Jean Malnati Brain Tumor Institute, Northwestern University, Chicago, IL 60611, USA; stephen.magill@nm.org (S.T.M.); matthew.tate@nm.org (M.C.T.); 4 Department of Pathology, Mayo Clinic, Jacksonville, FL 32224, USA; horbinski.craig@mayo.edu; 5 Department of Neurological Surgery, Northwestern University, Chicago, IL 60611, USA; joshua.rosenow@nm.org (J.M.R.); jason.k.hsieh@nm.org (J.K.H.)

**Keywords:** brain tumor-related epilepsy, electrocorticography, epilepsy, glioma, glioneuronal tumors, lesional epilepsy, meningioma, seizures

## Abstract

**Simple Summary:**

Brain tumors are commonly associated with seizures, which can seriously impact quality of life. This review examines surgical management strategies and seizure outcomes across different tumor types. Advances in surgical techniques and adjunctive tools that improve localization and resection of epileptogenic cortex are highlighted. In addition, the review discusses developing technologies currently being used to improve outcomes in tumor-related epilepsy and summarizes ongoing clinical trials and research efforts. Overall, it emphasizes a comprehensive surgical approach that balances oncologic goals with optimization of seizure outcomes.

**Abstract:**

Brain tumor-related epilepsy (BTRE) is a common and debilitating symptom of central nervous system (CNS) tumors. The epileptogenic zone, defined as cortex responsible for seizure generation, is located at the peritumoral region for most tumors, and lower-grade intrinsic brain tumors have the highest seizure incidence. Surgery is often the most effective treatment for the reduction in seizures in BTRE. However, surgical decisions have historically often been made exclusively for oncologic reasons, with less emphasis on seizure control. Surgical approaches for all tumor types are reviewed, highlighting relevant risk factors. Adjunctive tools during surgery, such as intraoperative electrocorticography (ECoG), may help identify and remove surrounding brain areas which are epileptogenic. Minimally invasive surgery is also gaining traction, given its utility in treating seizures deep-seated tumors. This review explores epileptogenic brain tumors, surgery for BTRE, and emerging strategies to better achieve seizure control.

## 1. Introduction

Incidence of brain tumor-related epilepsy (BTRE) varies by tumor type, with lower grade lesions carrying the highest seizure risk. Table 1 summarizes seizure likelihood by tumor classification. Tumor location and additional preoperative risk factors for tumoral epilepsy are also characterized by tumor type.

Low-grade epilepsy-associated tumors (LEATs), such as gangliogliomas and dysembryoplastic neuroepithelial tumor (DNET), are highly epileptogenic lesions benefitting from surgery for drug-resistant epilepsy (DRE); antiseizure medications (ASMs) are discontinued in up to 50% of cases [21]. Incidence of glioma-related epilepsy (GRE) corresponds to World Health Organization (WHO) grade and IDH mutational (IDHmut) status [22]. Seizures occur in 57–77% of low-grade gliomas (LGGs) versus 30–49% of high-grade gliomas (HGGs) [1] Although grade 2–4 gliomas generally achieve transient epilepsy control with resection, 56% of patients experience seizures postoperatively [23]. Implementation of intraoperative electrocorticography (ECoG), extended lesionectomies, and supramaximal resection (SMR) may improve these outcomes [24,25,26]. For meningiomas, resection remains the mainstay treatment for seizures. Approximately 30% of patients are diagnosed with epilepsy preoperatively and >30% of those patients continue to have seizures postoperatively [16,27]. In comparison, cerebral metastases have lower overall rates of epilepsy (15–20%) [28]. These rates depend on the cell of origin, as lung adenocarcinoma and melanoma are considered the most seizure-prone metastases [29,30]. The rapid growth of cerebral metastases, leading to hemorrhagic components, further increases seizure risk [31].

This review outlines the most epileptogenic CNS tumors and discusses various surgical approaches used to achieve seizure freedom. In addition, we highlight emerging technologies, such as artificial intelligence, molecular profiling, and new imaging methods. The future combination of these strategies will provide more precise surgical care. We lastly reiterate the need for a collaborative approach to both BTRE treatment and future research studies.

## 2. Medical Management of Tumor-Related Epilepsy: An Overview

Seizures are a common presenting symptom in patients with brain tumors. For patients who present without having had a seizure, SNO (Society for Neuro-Oncology and EANO (European Association of Neuro-Oncology) guidelines state that empiric ASM prophylaxis is not recommended. Both preoperative and postoperative prophylaxis are common, although evidence of their benefits remains mixed [32].

BTRE management is nuanced, as most cases must consider interactions with oncologic therapy. Furthermore, randomized clinical trials investigating the efficacy of ASMs in BTRE are scarce. First-generation ASMs (e.g., phenytoin or carbamazepine) are often avoided due to enzyme-inducing effects and side effects (e.g., thrombocytopenia and hepatotoxicity from valproic acid). Such drug profiles support use of newer generation ASMs, such as levetiracetam or lacosamide, for postoperative seizures. Lamotrigine may also be considered, although its need for prolonged titration limits its use as first-line therapy [33].

Prophylactic use of ASMs in patients with a brain tumor without a prior seizure does not reduce risk of seizures in the long-term, although physicians commonly prescribe ASMs to seizure-naïve brain tumor patients in clinical practice. Furthermore, it is well known that patients are at increased risk of seizures after intracranial surgery; however, use of prophylactic ASMs in prospective studies has been shown to not significantly reduce the likelihood of postoperative seizures [34]. However, these studies are not classified as Class 1 evidence and are generally underpowered. In general, patients with BTRE require indefinite ASMs, although some with >2 years of seizure freedom and no tumor progression may consider discontinuing them [35]. Finally, there is increasing interest in characterizing the epileptogenicity of tumors based on new imaging techniques, molecular profile, and topography; however, the evidence does not yet support the use of these factors in ASM management. Table 2 provides a summary of commonly used ASMs for tumoral epilepsy.

## 3. Surgical Approaches for Tumor-Related Epilepsy: An Overview

As patients with BTRE often have parallel, but unique treatment goals (i.e., oncologic treatment and seizure control), management of BTRE can benefit from the combined efforts of a multidisciplinary team. Seizure frequency often improves with treatment of the tumor itself via cancer-targeted strategies (i.e., surgical resection, radiation, and systemic chemotherapy). Surgical timing depends on tumor grade. WHO grade 1 tumors (e.g., glioneuronal tumors) are often resected for epilepsy purposes; however, in rare circumstances, resection may be necessary for other reasons, including progressive growth, mass effect, neurological symptoms, compression of critical structures, and reasons other than oncologic control. Additionally, the diagnosis of a WHO grade 1 tumor is not always definitive preoperatively; some tumors initially suspected to be low-grade may contain higher-grade components or demonstrate unexpected malignant potential. On the other hand, WHO grade 2–4 infiltrative gliomas, suspected higher-grade meningiomas, and symptomatic brain metastases usually undergo resection for oncological reasons. Rates of seizure freedom after tumor resection based on tumor type are presented in Table 3.

Various surgical strategies and tools exist to enhance seizure freedom in BTRE. Intraoperative electrocorticography (ECoG), a technique that records electrical activity directly from the surface of the cerebral cortex, is used to identify regions generating seizures and can guide resection. It is often employed for both grade 1 and high-grade lesions to better define the epileptogenic region and enhance seizure freedom [25]. However, many prior reports have failed to stratify ECoG outcomes according to tumor histopathology, grade, and location. In addition to resection, minimally invasive techniques have emerged as a new treatment option for BTRE. Laser interstitial thermal therapy (LITT) is one modality, which uses a light-emitting catheter to deliver thermal therapy to epileptogenic tissue [50]. It can offer seizure control and reduces tumor burden, especially in patients with deeply located tumors [51]. When determining a patient’s surgical candidacy, one variable is tumor localization relative to eloquent cortex. Intraoperative neurophysiological techniques (e.g., awake mapping) are routinely used to allow maximal resection while reducing the risk of functional deficit [52]. Across all tumors, EOR broadly corresponds to a greater likelihood of seizure freedom [53]. In summary, all available adjuncts and alternative surgical strategies, such as LITT, should be considered in the appropriate patients to reduce both risk of functional deficit and the risk of epilepsy.

## 4. Tumor-Related Epilepsy: Overview by Tumor Type

### 4.1. Long-Term Epilepsy-Associated Tumors

LEATs are WHO grade 1 tumors with predominant cortical localization, low growth rate, and low malignant potential, often presenting in adolescents as pharmaco-resistant focal epilepsy that is responsive to surgery [54]. Among LEATs, glioneuronal tumors (GNTs), including DNETs and gangliogliomas, are commonly epileptogenic and require surgery. Of all surgeries for DRE across 12 European countries, DNET and ganglioglioma were identified in 6% and 10% of histopathological examinations, respectively [55].

Gangliogliomas are slow-growing, well-differentiated GNTs composed of dysplastic ganglion cells and neoplastic glial cells. They account for less than 1% of all brain tumors, most frequently affecting people between age 9 and 25 [56]. While ganglioglioma can grow anywhere in the CNS, most are located supratentorially in the temporal lobe (70–80%) [6].

DNETs, on the other hand, are rare, benign, multinodular temporal lobe tumors. They present with seizures in adolescence, most commonly at ages 10–14 [57]. Pathologically, they are hallmarked by columns of axonal bundles situated orthogonally to the superficial cortex. Seizures occur in 94% of cases [1].

### 4.2. Infiltrating Gliomas

#### 4.2.1. Low-Grade Infiltrating Gliomas

Infiltrating LGGs (WHO grade 2) include IDHmut astrocytoma and IDHmut or 1p/19q-codeleted oligodendroglioma. Like other tumor types, glioma-related epilepsy significantly impacts quality of life [58]. Seizure prevalence exceeds 57% and is often the initial presenting symptom [1]. This is particularly true for IDHmut tumors and gliomas with oligodendrocytic components, likely due to their cortical predilection [22,40]. Temporal and insular tumors may grow large without causing functional deficits, particularly in the nondominant hemisphere. Thus, seizures may drive clinical symptoms rather than focal deficits [59]. Changes in seizure status often are an early indicator of tumor progression [10,40].

#### 4.2.2. High-Grade Infiltrating Gliomas

Infiltrating HGGs (WHO grade 3–4) are primarily IDH-wildtype (IDHwt) glioblastoma, yet also include IDHmut, 1p/19q codeleted oligodendroglioma, IDHmut astrocytoma, H3 K27-altered diffuse midline glioma, and H3 G34-mutant diffuse hemispheric glioma. Preoperative seizures occur in approximately one-third of HGGs, often in tumors localized to frontal/temporal lobes [14,15]. Interestingly, smaller tumor volumes have been associated with more frequent seizures [59]. Postoperative seizure recurrence, especially after >6 months, has been identified as an independent predictor of tumor recurrence [15,46].

### 4.3. Meningiomas

Meningiomas are slow-growing tumors originating from arachnoid cap cells of the meninges. Approximately one-third of patients present with seizures [60]. Invasive, higher-grade, and tuberous sclerosis 2-mutated meningiomas have been associated with increased seizure risk [61]. Furthermore, key predictors include peritumoral edema, male sex, and intratumoral calcifications [16,62]. While approximately two-thirds of patients presenting with a seizure will be able to discontinue seizure medications and remain seizure-free provided their tumor is adequately treated, more than one-third of patients with preoperative seizures will require long-term ASMs after resection [27].

### 4.4. Cerebral Metastases

Brain metastases are the most common adult intracranial tumors, occurring in 20% of patients with solid malignancies, with melanoma and lung cancer being the two most common tumor types metastasizing to the brain [30]. They are likely the most seizure-prone metastases [29]. Seizure presence has been linked with worse overall survival (OS) [63]. At the population-level, 10% of patients (≥66 years of age) with brain metastases develop seizures [20].

## 5. Seizure Outcome Classification

Engel classification (I–IV) is the most common method for assessing postoperative seizure outcomes. Class I indicates complete seizure freedom or nondisabling focal seizures. Class II–IV define higher seizure burdens, from rare disabling seizures (II) to no meaningful improvement (IV). Some studies report seizure outcomes with the more granular International League Against Epilepsy (ILAE) classification scale (I–VI). Although these systems are widely used in epilepsy literature, many studies report outcomes without standardized criteria, limiting cross-study comparability.

## 6. Surgical Resection and Seizure Outcomes

### 6.1. Surgery for Long-Term Epilepsy Associated Tumors

LEATs are typically resected for seizure control rather than oncologic reasons due to low malignant potential. Gross total resection (GTR) leads to optimal seizure freedom, with reported rates of 80%, 78%, and 76%, one, two, and five years after surgery, respectively [21]. In a systematic review across various WHO grade 1 and 2 lesions, GTR resulted in a higher likelihood of seizure freedom compared to subtotal resection (STR) ≥ 6 months after surgery (79% versus 43%). In patients with temporal lobe epilepsy and an associated WHO grade 1 lesion, GTR plus hippocampectomy and corticectomy further improved seizure outcomes [53]. Positive postsurgical prognostications for seizure control are surgery directed at the temporal lobe, young age at surgery, and shorter course of epilepsy [21].

### 6.2. Surgery for Infiltrating Gliomas

The optimal resection strategy for infiltrating gliomas is maximally safe resection, including consideration of supramaximal resection (SMR), when possible. Gross total resection (GTR) describes the removal of the entire enhancing region of a glioma or, in non-enhancing infiltrative gliomas, the FLAIR-hyperintense volume. A limitation of GTR is that glioma cells persist beyond the visible tumor margins, often into regions that appear radiographically normal. These infiltrative cells may not disrupt the Blood–Brain Barrier and therefore do not enhance on contrast MRI, making them difficult to identify and remove surgically [64]. In addition, some infiltrating gliomas, such as lower-graded IDHmut gliomas, have minimal contrast enhancement [65]. To address this limitation, supra-total/maximal/marginal approaches have been advocated. For example, supra-total surgery has been proposed to remove tissue beyond MRI abnormalities up to eloquent boundaries, regardless of whether the tissue appeared healthy [66].

Considering the growing body of data supporting the removal of tissue beyond the T1-enhanced margin, the Response Assessment in Neuro-Oncology (RANO) group has defined supramaximal surgery as resection “beyond the contrast-enhanced tumor borders” [67]. In this report, glioma patients with SMR experienced longer survival than those who underwent STR/GTR. Additionally, SMR has shown a positive impact on glioma-related epilepsy [24]. The hyperexcitable tissue leading to seizures in gliomas is located outside the tumor margin, at the peritumoral region, which may not demonstrate T1-enhancement [68]. Thus, the tissue with the greatest seizure risk may remain after GTR. Data of postoperative glioma-related epilepsy control after SMR is limited, but a subset of retrospective studies has reported positive outcomes [69].

#### 6.2.1. Resection for Low-Grade Infiltrating Gliomas

SMR for LGGs is associated with improved survival and seizure freedom [70]. In one series, 100% of patients were seizure-free ≥ 8 years post-SMR, with most reducing ASMs [71]. Other studies show SMR, when guided by intraoperative mapping or navigated transcranial magnetic stimulation (nTMS)-ECoG, improves seizure control at 3 months after surgery compared to GTR [24]. However, there remains a need for studies with longer, clinically relevant timepoints. EOR strongly predicts seizure freedom in LGGs, with GTR being a powerful independent predictor [39,40,53]. However, GTR is not always feasible due to infiltration of nearby eloquent regions. Receiver operative characteristic analysis suggested optimal glioma-related epilepsy outcomes with EOR of ≥91% and ≤19 cc residual tumor volume [72]. A drawback to most studies is a lack of stratification based on molecular signature.

#### 6.2.2. Resection for High-Grade Infiltrating Gliomas

Surgery is the first-line treatment for HGGs, with evidence suggesting SMR improves survival over GTR [70]. One study found 100% seizure control with GTR plus anterior temporal lobectomy (ATL) versus 50% seizure control with GTR alone in temporal lobe glioblastoma [73]. While less studied than LGGs, HGGs also gain seizure control through GTR/STR. One study showed >75% Engel I outcomes for HGG at 1 year, however EOR did not increase the likelihood of postoperative GRE control [15]. In grade 3–4 insular gliomas, 81% of patients achieved Engel I or II at follow-up (median, 21 months) [74]. Across grade 2–4 gliomas, 1-year seizure freedom has been reported least favorable (26%) in grade 4 glioma [42]. Although surgery provides an element of glioma-related epilepsy control, the surgical approach for HGGs, as well as most LGGs, revolves around oncologic management.

### 6.3. Surgery for Meningiomas

Surgery is recommended for symptomatic meningiomas, especially in younger patients with large, accessible lesions. Stereotactic radiosurgery (SRS) may be preferred for certain small or difficult-to-access lesions, particularly in high-risk surgical candidates. In meningioma-related epilepsy, seizure freedom at ≥6 months is achieved in 67% of patients after resection, but 12% will develop new seizures [16]. At 5-year follow up, seizure freedom is seen in 60% of patients with, and 90% without, preoperative seizures [17,75]. Predictive factors for postoperative seizures include higher WHO grade, preoperative seizures, tumor > 3 cm diameter, postoperative epileptiform discharges on EEG, and surgical complications [17,47,75,76]. The need for temporal lobe retraction, particularly with basal or subtemporal surgical approaches, also increases seizure risk in meningiomas. The STAMPE2 score has been developed to identify individuals at high-risk for developing epilepsy [76]. The ongoing STOP‘EM trial, scheduled to conclude in 2027, will hopefully shed more light on whether prophylactic ASM management decreases the rate of postoperative seizures in meningioma patients [77].

### 6.4. Surgery for Cerebral Metastases

The most common treatment of brain metastases is stereotactic radiosurgery, followed by surgery, then fractionated radiotherapy, and LITT gaining popularity. Surgical resection is supported in patients with good performance status, who have well-controlled systemic disease and isolated brain metastases, or who have one or very few large, symptomatic lesions, particularly in close proximity to each other. Total en bloc resection is preferred to limit recurrence [78]. Often multiple lesions are present; therefore, imaging alone cannot determine the epileptogenic metastasis. In these cases, seizure semiology may aid in localization of the symptomatic lesion [79]. After surgery, seizure control (Engel I) was achieved in 88–93% of patients at follow-up, although the link between rate of seizure freedom and EOR is unclear [19,31,80]. As with other brain tumors, prophylactic ASMs do not prevent seizures, and risk factors for postoperative seizures include preoperative seizures, tumor recurrence, and multiple surgeries [29,31,80].

## 7. Timing of Surgery

In general, surgery should be performed when the patient is medically stable and in optimal condition to tolerate resection. The timing of surgery depends on the suspected tumor type and grade, as well as the patient’s responsiveness to antiseizure medications. For clinically stable patients, surgical timing follows the considerations outlined for each tumor type. However, when patients present in status epilepticus, urgent surgical intervention may be warranted.

When patients are in status epilepticus—defined as recurrent seizures without recovery between events—the initial priority is typically to achieve seizure control before proceeding to resection. Although exceedingly rare, if the patient develops super-refractory status epilepticus, characterized by seizures persisting ≥ 24 h despite anesthesia or recurring during weaning of anesthesia, emergent surgical intervention may be warranted.

## 8. Seizure Control with Extended Lesionectomies

Lesionectomy (i.e., removal of the tumor alone) may not fully control seizures, depending on tumor type. Possible reasons include epileptogenic activity in surrounding “normal” tissue, involvement of perilesional cortex in the seizure network, or coexisting cortical malformations [81].

Epileptogenicity in low-grade lesions often extends beyond the lesion and can be observed as epileptiform activity in surrounding cortex recorded via intracranial electrodes. In patients with temporal lobe low-grade tumors, such as LGGs, DNETs, and gangliogliomas, extended lesionectomy (GTR plus hippocampectomy/ATL) achieves superior seizure freedom [53]. In one study evaluating the use of intraoperative ECoG for temporal lobe LGG surgery, 87% of epileptiform discharges had been localized to the anterior part of the temporal lobe with the remainder in the medial and posterior temporal lobe. GTR plus ATL provided Engel class I or II seizure control in 93% of these patients [26]. In comparison, frontal LGGs showed discharges in adjacent tissue or interestingly, the temporal lobe; SMR provided relief of both discharges [26]. With the current available data, extended resection beyond a lesionectomy should be considered in low-grade tumor cases and sometimes may require evaluation with intracranial electrode recordings to determine the full extent of the epileptogenic zone.

## 9. Adjunct Techniques

### 9.1. Electrocorticography-Guided Resective Surgery

ECoG, recording directly from the cortical surface, can guide surgeons in identifying and resecting an epileptogenic focus. While useful, this technique can be limited due to anesthetic considerations and the brief duration of recording. Furthermore, epileptiform discharges arising from eloquent tissue may not be amenable to resection. This is especially relevant in temporal lobe tumors where frequent spikes may originate from functionally critical areas, such as the hippocampus. It is important to note that, due to selection bias, seizure outcomes may be skewed in studies by patients having complex epilepsy, as evidenced by the decision to recommend ECoG. Prospective data, which would address this methodological limitation, is lacking; therefore, current evidence should be interpreted with caution.

Some studies suggest that ECoG may improve seizure freedom for temporal lobe gangliogliomas, while other studies show no benefit [82,83,84]. Given the limited data and small sample sizes, more research is needed to clarify ECoG’s role for this tumor type.

ECoG has also been studied in the context of surgical resection of DNETs. One pediatric study reported Engel class I outcomes in 21 out of 22 children after ECoG-guided extended resection, with another showing seizure freedom up to 44 months of follow-up [85,86]. Just as with gangliogliomas, there is conflicting evidence on the utility of this technique; in a systematic review of 910 patients, seizure freedom rates were similar with and without ECoG guidance (75% vs. 77%) [36]. One common consideration in treating DNETs is the presence of surrounding focal cortical dysplasia (FCD), which may contribute to the overall epileptogenic network. Presence of FCD is variable across patients with DNETs. ECoG may be valuable for assisting resection in these cases, as epileptogenic regions outside of the main tumor may be identified. Given the evidence of ECoG’s benefit for all DNETs is controversial, it may instead be better to focus its use on this targeted patient subset at this time [87,88].

ECoG studies in WHO grade 1–2 gliomas report differing levels of benefit; while some suggest improved seizure outcomes with its use, others find no additional advantage [26,39]. These studies have the limitation of not stratifying seizure freedom rates based on tumor grade, pathology, and location. Interestingly, in cases where a lesion is associated with FCD, the presence of epileptiform discharges on ECoG post-resection does not significantly impact seizure outcome [25]. That is to say, not all discharges detected on ECoG need to be resected for a patient to become seizure-free with FCD. Still, a meta-analysis suggests ECoG might improve seizure control in FCD without an associated tumor [89]. Additionally, ECoG can help detect intraoperative seizures, a known risk during LGG surgery [90].

In addition to ECoG, stereoelectroencephalography (SEEG) can be used to localize the epileptogenic zone, particularly for grade 1 lesions with unclear seizure-onset zones or deep-seated tumors. Unlike ECoG, SEEG involves the implantation of depth electrodes and allows for extended, 3-dimensional recording in awake patients. SEEG can more accurately localize the seizure-onset zone with cortical recordings and guide tailored resections in complex cases [91].

More high-quality research is needed to clarify the value of ECoG and SEEG for improving seizure outcomes for BTRE, especially in well-defined patient subsets, given current data is underpowered.

### 9.2. Awake Mapping

Awake craniotomy with intraoperative cortical stimulation mapping is the gold standard approach for the removal of tumors in eloquent regions [52,92]. It serves a distinct yet complementary role to ECoG for tumor resection. Certain anesthetic agents suppress neuronal activity and in consequence, compromise detection of epileptiform discharges [93]. Therefore, anesthesia protocols should be modified when there is a plan for intraoperative ECoG and/or awake mapping. ECoG should be used during cortical stimulation to monitor for afterdischarges to confirm that clinical findings are due to stimulation alone rather than afterdischarges or seizures. The role of preoperative ECoG in awake mapping for patients with BTRE remains uncertain. Nonetheless, when epileptiform discharges are identified, the surgeon may elect to reduce stimulus intensity in the affected region and/or defer mapping of that area until later in the procedure, thereby minimizing the risk of seizures that could preclude completion of mapping.

Given awake surgery has enhanced EOR in eloquent gliomas, it is feasible that seizure freedom follows in the same vein [94]. 100% of patients undergoing awake mapping for glioma-related epilepsy were seizure-free at >1 year in a prospective analysis [95]. In a retrospective sample of glioma-related epilepsy patients (n = 41), 81% showed ILAE class 1 or 2 at 6 months. However, comparative analysis of awake and asleep surgery for glioma-related epilepsy had similar postoperative seizure rates at <1 year, although this study did not control for EOR [96].

## 10. Laser Interstitial Thermal Therapy (LITT)

LITT is a minimally invasive treatment that uses a laser-emitting catheter to deliver focused thermal energy, allowing for precise laser ablation of lesions in the brain. Although there is promising data supporting the ability of LITT to increase the likelihood of seizure freedom for various epilepsy etiologies, including hypothalamic hamartoma, cortical heterotopia, and tuberous sclerosis; there is limited data focused on outcomes in patients with BTRE [97]. A single-institution study reported patients with LEATs who were treated with LITT achieved comparable seizure freedom and lower morbidity when compared to open tumor resection [98,99]. LITT is useful for ablating deep-seated lesions while sparing overlying cortex, reducing surgical morbidity and length of stay. This idea is supported by an 826-patient meta-analysis reporting a low rate of adverse events after LITT in HGGs, LGGs, and brain metastases. It was found that postoperative seizures (at any time after ablation) occurred in only 6% of cases [100].

LITT has potential for treating radiation necrosis, also known as post-treatment radiation effect (PTRE), which can cause neurologic complications, including seizures. In one study, 10% of SRS patients developed PTRE, and of these, 8% developed seizures [101]. Another detected shifts toward fewer seizures within 90 days of LITT compared to medical management for radiation necrosis [102].

## 11. Developing Technologies

**Figure 1:**
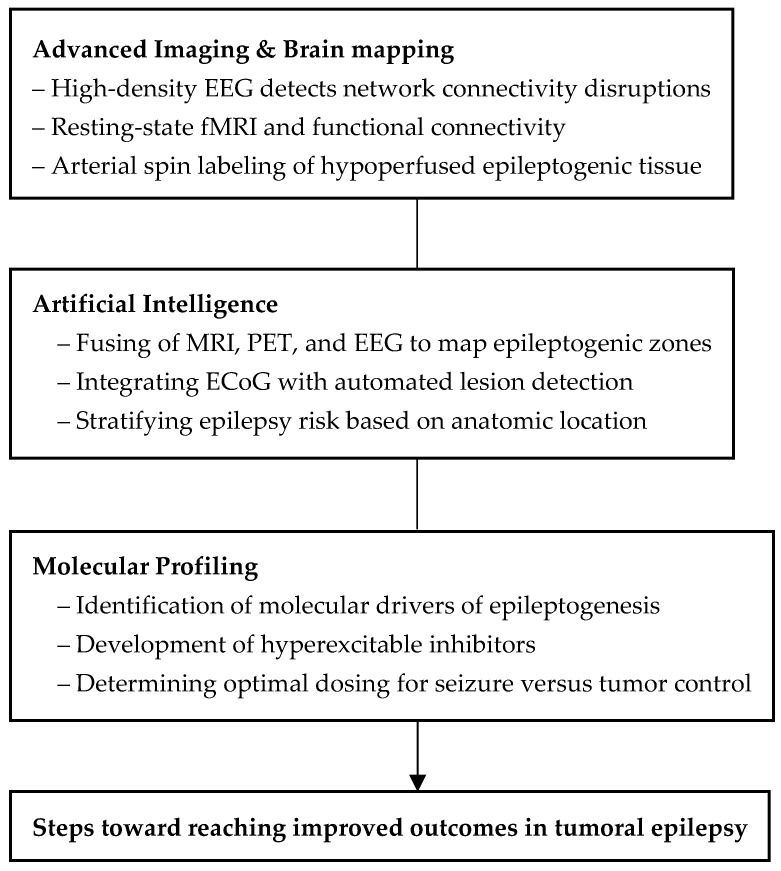
The intersection of three advancing technological domains is positioned to enhance both seizure and oncologic control of brain tumors.

There are several tools that may help better characterize tumors’ epileptogenic potential: artificial intelligence (AI), new imaging modalities, and molecular biomarkers. Integrating these into the clinical workflow may improve surgical outcomes. Figure 1 highlights emerging technologies aimed at improving outcomes in tumoral epilepsy.

### 11.1. Augmenting Surgical Planning via Artificial Intelligence

Ongoing research is examining how AI may augment multiple components of epilepsy care, including neuroimaging, EEG, and medical devices [103]. AI can be useful for identifying seizure risk based on tumor location, histopathology, and demographic characteristics; one study employed a generalized additive model to evaluate how tumor topography influences this risk [104]. Additionally, AI has the potential to assist in preoperative detection of FCD, especially in cases where human readers are unable to localize dysplastic parenchyma on MRI [105]. Given that intraoperative ECoG can also help identify the irritative zone and help guide resection, there remains a need to evaluate its combined use with AI lesional detection in maximizing resection. Other AI models have demonstrated similarly improved detection of the seizure-onset zone in cases where MRI is inconclusive [106].

### 11.2. Emerging Brain Mapping Techniques

In addition to AI and ECoG, other technologies aim to enhance detection of epileptogenic regions associated with brain tumors. High-density EEG provides a more detailed understanding of network-level disruptions associated with gliomas, which directly relates to the mechanism of epileptogenesis. This technology measures several parameters related to network function, and perturbations in the balance between these parameters can result in increased seizure activity. A network topology analysis using EEG found that glioma-related epilepsy had higher global and local efficiencies and shorter path length, facilitating quicker seizure spread and lower threshold of seizure activity [107]. Imaging modalities, such as magnetoencephalography (MEG), dual-modality positron emission tomography (PET)/near-infrared fluorescence, and arterial spin labeling, have also been used to detect epileptogenic tissue for operative planning. Resting-state functional MRI will be increasingly adopted in clinical practice, as 3 neuroradiologic societies have created an acquisition/pre-processing protocol for BTRE [108]. These methods have been associated with improved seizure outcomes, high accuracy, and greater sensitivity compared to traditional methods [109,110,111].

### 11.3. Molecular Profiling in Predicting Epileptogenicity

Molecular profiling is another tool that can help to predict epileptogenicity, as specific gene expression (e.g., IDHmut) has been associated with greater seizure frequency in glioma [22]. Our understanding of the mechanism underlying increased seizure activity in the setting of IDHmut continues to grow, but at a basic level, there is a synergistic relationship between the hyperexcitable environment and tumor growth rate. Structurally similar to glutamate, it is hypothesized the byproduct of IDHmut gliomas, D-2-hydroxyglutarate, promotes seizure activity by activating excitatory channels or promoting an altered metabolomic tumor profile [112,113]. MicroRNAs are also proving to be important prognostic indicators; for example, expression of miR-1290 may be associated with greater seizure susceptibility in glioblastoma [114]. These expected changes associated with molecular biomarkers assist in postoperative management. For example, gangliogliomas with BRAF V600E mutation may exhibit seizure relapse after resection [115]. BRAF inhibitors have decreased seizure burden in animal models but currently do not have blood-brain penetrability for use as a potential ASM-adjunct in clinical practice [116]. On the other hand, a phase 3 trial of IDH inhibitors have shown to promote seizure control in infiltrating gliomas [117]. Molecular changes, which are often not available until postoperative pathology is reviewed, must be interpreted in the context of the associated imaging, EEG, and clinical context to fully inform surgical decisions. Advances in Ramen spectroscopy may provide some molecular information intraoperatively, allowing incorporation of these data into surgical strategy [118].

## 12. Clinical Trials

Despite significant progress in surgical and pharmacological management, prospective clinical trials in BTRE remain scarce, with most available data derived from retrospective studies. However, several recent trials have begun to address seizure control across specific tumor subtypes.

### 12.1. Low- and High-Grade Gliomas

Recent trials have highlighted the use of antiseizure therapies in managing gliomas. STING is an ongoing trial examining the efficacy of levetiracetam versus valproic acid as first-line therapy for tumor-related epilepsy, with six-month seizure freedom as the primary outcome. Unlike many of the tumor therapy trials, STING directly evaluates seizure control. Outcomes have not yet been reported (NCT03048084). Another trial, SPRING, examined the utility of prophylactic levetiracetam in seizure-naïve glioma patients who were undergoing surgery [119]. Unfortunately, the trial was underpowered due to poor accrual, and the results showed no difference in 12-month seizure risk between treatment and control groups. Future trials should incorporate both pharmacologic and surgical interventions in treating gliomas with seizure freedom as a primary endpoint. Data from the RANO working group indicates that seizure control correlates with disease progression in glioma, further supporting the relevance of this outcome in neuro-oncology [120].

### 12.2. Meningioma

The STOP’EM trial is an ongoing multicenter randomized trial evaluating whether two-week prophylactic levetiracetam treatment reduces seizure risk within 1 year of resection of seizure-naïve meningioma (NIHR129748). Results are not yet available but are expected to clarify whether routine short-term prophylaxis is warranted in this population [77].

Similarly, the LeviTaTe pilot trial is assessing the efficacy of short-term levetiracetam prophylaxis for seizure control in patients undergoing resection of a supratentorial brain tumor (NCT05897658). This study does not focus on a specific tumor subtype, but further highlights that there is active work being carried out to generate evidence for seizure prophylaxis in brain tumor surgery.

Together, these trials highlight the increasing interest in the standardized, prospective evaluation of seizure control in patients who suffer from brain tumors—an area that has previously heavily relied on observational data.

## 13. Conclusions and Future Perspectives

Seizures are a complication of various brain tumors. In general, tumors of lower malignant grades have the greatest potential to become epileptogenic. WHO grade 1 brain tumors with low potential for malignant transformation often opt for surgery to control seizures, whereas grade 2–4 lesions require maximal resection within functional boundaries to mitigate malignant progression.

Despite the approach, greater EOR across all tumor types, with potentially the exception of brain metastases, leads to better seizure control [19]. Medically refractory LEATs (e.g., DNET or ganglioglioma) demonstrate good seizure control after surgery, leading to about half of patients discontinuing ASMs [21]. For gliomas, seizures may persist; although if patients are initially seizure-free after resection, recurrence may signal tumor progression and/or recurrence [15,40,46]. The heterogeneous nature of brain tumors—even among those of the same type and grade—poses significant challenges for conducting systematic studies on seizure outcomes. Nonetheless, the profound impact of seizures on patients’ quality of life underscores the importance of continued investigation. Future investigations will require robust multi-institutional collaboration and the aggregation of large, standardized datasets to elucidate optimal surgical strategies that address both oncologic control and seizure management.

Surgical resection is often the most effective treatment for BTRE. In tumors with DRE, surgery is frequently the only modality to achieve seizure freedom. However, referrals are often delayed, increasing morbidity. A collaborative, multidisciplinary approach across neurology, neurological surgery, epilepsy, and neuro-oncology becomes beneficial in expediting referrals and paving a clinical pathway to identify appropriate surgical candidates.

Moving forward, the surgical landscape of BTRE will continue to shift toward the utilization of functional mapping techniques. These approaches are most used for infiltrating gliomas where tumors are diffusely invasive with poorly defined margins. Better characterization of the electrophysiologic signature across tumor types and their functional influence on nearby tissues may tailor specific resective boundaries. If temporospatial integrations are different from one lesion to the next, which seems to be the case, different classification schemes may be needed to identify epileptogenic zone(s) and guide resection. Optimal localization of the epileptogenic zone depends on the integration of neurophysiologic data (e.g., ECoG or MEG) with functional imaging modalities (e.g., PET or fMRI). However, practical constraints—particularly limited time prior to surgery—often hinder the ability to acquire these additional data points. In addition, their precise utility in surgical decision-making remains uncertain. Nonetheless, serial functional imaging enables tracking of large-scale cortical changes in BTRE. A better understanding of this evolving tumor connectome may allow us to anticipate ASM resistance and recommend earlier surgical intervention.

There is an emerging imperative to conduct multicenter, prospective studies with long-term outcomes on BTRE. It is now well-established that the hyperexcitable neuronal-tumor interface plays an integral role in the invasiveness and proliferation of malignant lesions, such as gliomas and cerebral metastasis. This hyperexcitable network drives seizure activity [68]. HGGs integrate into and reshape neuronal circuits, disrupting macro-level neural connectivity [121]. These findings support a connectomal resection strategy for gliomas [122]. Maximizing the resection of tumors and tumor-associated epileptogenic foci while protecting eloquent tissue can enhance seizure control. The RANO group recently defined and summarized the potential benefits of SMR for survival in grade 2 IDHmut and grade 4 IDHwt gliomas, which may also have a benefit for BTRE. Effective removal of the hyperexcitable peritumoral cortex may contribute to this survival benefit. The definition of SMR is currently broad, and refinement of this definition will allow for systematic evaluation and comparison of seizure outcomes in this population [67]. Lastly, implementing ASM-tapering protocols, neurocognitive scales, and seizure classification systems (e.g., Engel or ILAE) are three other courses of action to enhance the impact of future BTRE trials.

In conclusion, lesional resection remains the cornerstone of treatment for epileptogenic tumors refractory to ASMs. Surgical strategies for BTRE are headed toward greater precision and integration of adjunctive technologies. The success of managing this challenging pathology ultimately depends on effective interdisciplinary collaboration.

## Figures and Tables

**Table 1:** Characteristics of epileptogenic brain tumors.

Brain Tumor	Seizure Prevalence	Preoperative Seizure	Topography	Seizure Type	Preoperative Risk Factors
DNET	83–100% [1]	92–100% [2,3]	Temporal (66%) Frontal (20%) [4]	Bilateral tonic clonic (32%) Focal (76%) Focal to bilateral tonic–clonic (6%) [2]	Location within temporal lobe or insula [5]
Ganglioglioma	66–97% [1]	64–97% [6,7]	Temporal (79%) Frontal (12%) [6]	Focal (67%) Bilateral tonic–clonic (33%) [8]	Supratentorial location, temporal lobe involvement [9]
Infiltrating Low Grade Glioma	57–68% [1]	69–90% [10,11]	Frontal (48%) Temporal (41%) [12]	Focal (42%) Focal to bilateral tonic–clonic (37%) Other (21%) [12]	Hypertension, no comorbidity [13]
Infiltrating High Grade Glioma	30–38% [1]	22–24 [14,15]	Frontal (46%) Temporal (33%) Parietal (16%) [15]	Focal (37%) Bilateral tonic clonic (35%) [15]	Focal impaired seizure type, tumor size, hemorrhagic component [15]
Meningioma	20–28% [1]	12–76% [16]	Convexity (33%) Falx or parasagittal (38%) [17]	Focal (48%) Bilateral tonic–clonic or mixed (36%) Bilateral tonic clonic (10%) [18]	Male, absence of headache, peritumoral edema, non-skull base location [16]
Cerebral Metastases	18–29% [1]	10–24% [19,20]	Frontal (31%) Parietal (19%) Temporal (14%) Occipital (12%) Cerebellar (23%) [19]	Focal aware (32%) Focal impaired (18%) Bilateral tonic–clonic (39%) [20]	Frontal lobe location, melanoma diagnosis, KRAS mutation (in lung carcinoma), intratumoral hemorrhage, prior radiotherapy [19]

**Table 2:** Antiseizure Medications (ASMs) and Key Considerations in Brain Tumor-Related Epilepsy (BTRE). [Presented in order of frequency of use]

Antiseizure Medication	MOA/Target	Practical Notes
Levetiracetam	SV2A modulation	First-line agent; minimal drug–drug interactions; generally well tolerated; Monitor for mood changes (e.g., irritability) more commonly seen in setting of frontal lobe tumors
Lacosamide	Enhance slow inactivation of voltage-gated Na^+^ channels	Well tolerated; often tolerated with less side effects compared to LEV Monitor PR interval/risk of arrhythmias, dizziness, diplopia/ataxia at higher doses
Brivaracetam	High-affinity SV2A modulation	Similar interaction liability to LEV; consider if seizure free with LEV, but not tolerated due to mood side effects (~2/3 of patients report improved side effects)
Lamotrigine	Na^+^ channel blocking; decrease glutamate release	Slow titration required due to risk of rash/SJS/TEN; not for use as first-line agent given prolonged titration; good cognitive and mood profile Monitor for insomnia, dizziness/diplopia at higher doses
Valproic acid	Na^+^ channel blocking; T-type Ca^2+^ channel inhibition; GABA enhancement	Enzyme inhibition: CYP2C9 (strong); CYP2C19 (moderate); CYP3A4 (weak); UGT1A4/UGT2B7 (moderate) Monitor for hyperammonemia, transaminitis, thrombocytopenia and weight loss; Increased risk of thrombocytopenia with TMZ; May be an exclusion criterion for clinical trials Initial report of survival benefit in glioma not consistently reproduced
Clobazam	GABA_A_ receptor agonist	Adjunct therapy, although positive seizure response may allow wean of other ASM(s); low doses may be sufficient; Monitor for fatigue (initial side effects often resolve within weeks)
Zonisamide	Na^+^ channel blocking; T-type Ca^2+^ channel inhibition	Avoid in patients with history of nephrolithiasis or sulfa allergies; Monitor for weight loss, cognitive slowing, and metabolic acidosis
Perampanel	AMPA antagonist	Adjunct therapy; anti-glutamatergic tumor benefit shown in animal models; Monitor for irritability, aggression, and dizziness
Eslicarbazepine	Enhance slow inactivation of voltage-gated Na^+^ channels	Not well studied in BTRE; Na^+^ channel mechanism similar to carbamazepine/oxcarbazepine without same degree of enzyme interactions [moderate CYP2C19 inhibitor; mild CYP3A4 inducer Monitor for hyponatremia, renal dysfunction, dizziness
Gabapentin, Pregabalin	α2δ subunit of voltage-gated Ca^2+^ channels	Minimal effect on CYPs; minimal drug-drug interactions
***Avoid/limit with chemotherapy*:** Phenytoin, Phenobarbital, Carbamazepine, Primidone	Potent CYP induction	Enzyme induction reduces levels of oncologic agents (TMZ, TKIs, etc.) in addition to dexamethasone; Often listed as exclusion criteria for clinical trials
***Rescue*** ***Benzodiazepines***: intranasal Midazolam or Diazepam; PO clonazepam	GABA_A_ receptor allosteric modulator	1st-line for breakthrough seizures/status; intranasal are practical for outpatient use

ASM, antiseizure medication; LEV, levetiracetam; CYP, cytochrome P450; PO, per os; SJS, Stevens–Johnson syndrome; SV2A, synaptic vesicle protein 2A; TEN, toxic epidermal necrolysis; TKI, tyrosine kinase inhibitor; TMZ, temozolomide.

**Table 3:** Seizure freedom rates and predictive factors after tumor resection.

Brain Tumor	Rate of Seizure Freedom ^†^	Predictors of Postoperative Seizure Freedom	Predictors of Postoperative Seizures
Glioneuronal Tumors	^‡^ STR: 55% ^‡^ GTR: 87% [36]	GTR, focal to bilateral tonic–clonic seizures, seizure duration for <1 year, temporal lobe surgery, younger age [21,36]	Histopathology, concomitant cortical dysplasia, mesial temporal location [37]
Infiltrating Low Grade Glioma	STR: 49–60% GTR: 75–86% [38]	GTR, presurgical seizure control with ASMs, seizure duration for <1 year, frontal lobe location [12,39]	Focal seizures, preoperative seizures, mutational status, infiltrative pattern, histological subtype [12,39,40,41]
Infiltrating High Grade Glioma	STR/GTR: 26–77% [15,42]	GTR (if exclusively in temporal lobe), early seizure onset [43,44]	Temporal lobe location, preoperative epilepsy, molecular subtype [45,46]
Meningioma	^‡^ STR/GTR: 69% [16]	GTR, well-defined tumor margins, lower WHO grade [47]	Male, postoperative neurological deficits, postoperative complications, preoperative seizures, motor cortex location, non-skull base location, recurrence [18,48]
Cerebral Metastases	^§^ STR/GTR: 64% [49]	GTR (conflicting evidence) [19,31]	Checkpoint inhibitors, previous radiotherapy, older age, disease progression within CNS, parietal lobe, recurrence, multiple surgeries [19,29,31]

^†^ Seizure freedom defined as ILAE or Engel 1. ^‡^ Pooled estimate. ^§^ Calculated weighted average.

## Abbreviations

The following abbreviations are used in this manuscript:

AI	Artificial Intelligence
ASM	Antiseizure Medication
ATL	Anterior Temporal Lobectomy
BTRE	Brain Tumor-Related Epilepsy
CNS	Central Nervous System
DNET	Dysembryoplastic Neuroepithelial Tumor
DRE	Drug-Resistant Epilepsy
ECoG	Electrocorticography
EEG	Electroencephalography
EOR	Extent of Resection
FCD	Focal Cortical Dysplasia
GNT	Glioneuronal Tumor
GTR	Gross Total Resection
HGG	High-Grade Glioma
IDH^mut^	IDH-Mutant
LEAT	Low-Grade Epilepsy-Associated Tumor
LGG	Low-Grade Glioma
LITT	Laser Interstitial Thermal Therapy
MEG	Magnetoencephalography
nTMS	Navigated Transcranial Magnetic Stimulation
OS	Overall Survival
PET	Positron Emission Tomography
PRTE	Post-Radiation Treatment Effect
RANO	Response Assessment in Neuro-Oncology
SMR	Supramaximal Resection
SRS	Stereotactic Radiosurgery
STR	Subtotal Resection
WHO	World Health Organization
